# Receptor Characterization and Functional Activity of Pyrokinins on the Hindgut in the Adult Mosquito, *Aedes aegypti*

**DOI:** 10.3389/fphys.2020.00490

**Published:** 2020-05-21

**Authors:** Aryan Lajevardi, Jean-Paul V. Paluzzi

**Affiliations:** Laboratory of Integrative Vector Neuroendocrinology, Department of Biology, York University, Toronto, ON, Canada

**Keywords:** pyrokinin, G protein-coupled receptor, heterologous receptor functional assay, hindgut, motility, scanning ion-selective electrode technique, insect, disease vector

## Abstract

Pyrokinins are structurally related insect neuropeptides, characterized by their myotropic, pheromonotropic and melanotropic roles in some insects, but their function is unclear in blood-feeding arthropods. In the present study, we functionally characterized the pyrokinin-1 and pyrokinin-2 receptors (PK1-R and PK2-R, respectively), in the yellow fever mosquito, *Aedes aegypti*, using a heterologous cell system to characterize their selective and dose-responsive activation by members of two distinct pyrokinin subfamilies. We also assessed transcript-level expression of these receptors in adult organs and found the highest level of PK1-R transcript in the posterior hindgut (rectum) while PK2-R expression was enriched in the anterior hindgut (ileum) as well as in reproductive organs, suggesting these to be prominent target sites for their peptidergic ligands. In support of this, PRXa-like immunoreactivity (where X = V or L) was localized to innervation along the hindgut. Indeed, we identified a myoinhibitory role for a PK2 on the ileum where PK2-R transcript was enriched. However, although we found that PK1 did not influence myoactivity or Na^+^ transport in isolated recta, the PRXa-like immunolocalization terminating in close association to the rectal pads and the significant enrichment of PK1-R transcript in the rectum suggests this organ could be a target of PK1 signaling and may regulate the excretory system in this important disease vector species.

## Graphical Abstract

Schematic depicting pyrokinin target sites along the hindgut of *Aedes aegypti*. The *A. aegypti* PK2-R is expressed in the anterior hindgut region (ileum) and a pyrokinin-2 (PK2) peptide was shown to inhibit ileum motility, whereas the *A. aegypti* PK1-R is expressed in the posterior hindgut (rectum) indicating a pyrokinin-1 (PK1) peptide, such as *Aedae*CAPA-PK1, acts on the rectum, although its exact role remains unknown.

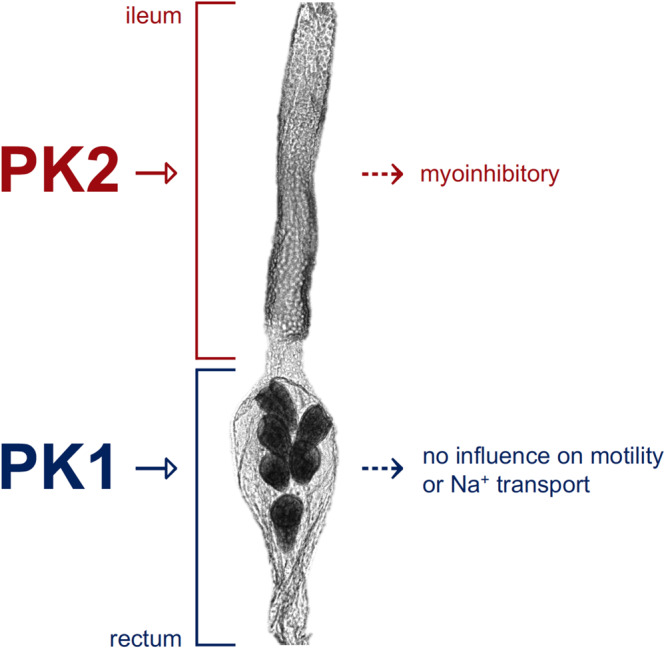

## Introduction

Neuropeptides regulate an array of physiological processes in insects, including feeding, metamorphosis, diapause, and reproduction (Nässel and Winther, 2010). One such group of neuropeptides is the pyrokinin (PK) family. The first member to be identified, leucopyrokinin, was isolated from the cockroach, *Leucophaea maderae*, based on its stimulation of hindgut motility (Holman et al., 1986). Subsequently, a neuropeptide isolated based on its induction of sex pheromone production in female corn earworm moths (*Helicoverpa zea*; Raina et al., 1989), so named pheromone biosynthesis activating neuropeptide (PBAN), was found to have the same carboxyl (C) terminus, FXPRL-NH_2_, and identification of the *pban* gene revealed additional encoded peptides with this conserved motif (Raina and Kempe, 1992; Sato et al., 1993). Related PKs have also been identified in other insects and shown to regulate cuticle melanization, pupariation, and feeding behavior (Matsumoto et al., 1990; Zdarek et al., 1997; Verleyen et al., 2004; Bader et al., 2007).

The existence of another PK subfamily with the highly conserved WFGPRL-NH_2_ C terminus was revealed with the characterization of the diapause hormone (DH) that regulates the onset of embryonic diapause in the silkworm *Bombyx mori* (Yamashita, 1996). The role of DH in diapause has been identified in some lepidopteran species (Xu and Denlinger, 2003), and more recently in *Locusta migratoria* (Hao et al., 2019). Other peptides with this conserved sequence have been identified in Diptera (Predel et al., 2004; Predel et al., 2010), but their physiological roles remain unclear. These neuropeptides, which are commonly referred to as tryptopyrokinins (Veenstra, 2014), are classified as the pyrokinin-1 (PK1) type, while PBAN-related neuropeptides, characterized by their pentapeptide core motif (FXPRL-NH_2_), are considered to be the pyrokinin-2 (PK2) type (Jurenka, 2015; Ahn and Choi, 2018). Members of the PK1 subfamily are encoded by two genes in most insects. The first was characterized for *B. mori* and called *pban* (Kawano et al., 1992; Sato et al., 1993), which also encodes PK2 peptides. A homologous gene has been characterized in *Drosophila melanogaster*, termed *hugin*, expressed in a subgroup of neurosecretory cells within the subesophageal ganglion (Meng et al., 2002; Bader et al., 2007), however, this gene in *D. melanogaster* only gives rise to PK2 peptides (Nässel and Winther, 2010). The second gene in insects encoding PK1 is the *capability* (*capa*) gene, which was first identified in *D. melanogaster* (Kean et al., 2002; Baggerman et al., 2002), which encodes not only a PK1 but also two additional neuropeptides known as CAPA or periviscerokinins that influence the activity of insect Malpighian tubules (Kean et al., 2002; Pollock et al., 2004; Terhzaz et al., 2012; Sajadi et al., 2018). Homologous *capa* genes were subsequently identified across insect groups and found to be expressed predominantly in a pair of neurosecretory cells in the abdominal ganglia and a subset of neurons of the subesophageal ganglion (Predel and Wegener, 2006; Hellmich et al., 2014). PK-producing neurons localized in these ganglia have axons extending to perisympathetic organs, where peptides either act on the nervous system or are released into the hemolymph to exert their actions at peripheral targets (Choi et al., 2001; Hellmich et al., 2014).

Efforts to define PK signaling in target cells have identified G protein-coupled receptors that selectively bind PK1 or PK2 forms found in *D. melanogaster*, the African malaria mosquito (*Anopheles gambiae*), and the kissing bug (*Rhodnius prolixus*) (Cazzamali et al., 2005; Olsen et al., 2007; Paluzzi and O’Donnell, 2012). These studies were conducted with cell systems expressing putative PK receptors cloned from the insects of interest, as was accomplished for the yellow fever mosquito, *Aedes aegypti* (Choi et al., 2013). However, in this latter study, the PK1 and PK2 receptors (PK-Rs) were only tested using pyrokinins encoded by the *AAEL012060* (*hugin*) gene, whereas activity of PK1 encoded by the *AAEL005444* (*capa*) gene was not determined. Although no functions have yet been assigned for PK neuropeptides in mosquitoes or any other hematophagous arthropods, expression profiles of PK receptors in *R. prolixus* and the deer tick, *Ixodes scapularis*, show enrichment in the nervous system and reproductive tissues, and to a lesser extent, in the prothoracic glands and hindgut of *R. prolixus* (Paluzzi and O’Donnell, 2012; Gondalia et al., 2016).

Female *A. aegypti* are chief vectors of the chikungunya, dengue and yellow fever, and Zika viruses that are the causative agents of acute and chronic illnesses in humans globally (Kotsakiozi et al., 2017). Improving our understanding of mosquito biology and the regulation of underlying physiological processes by neuropeptides is imperative in order to develop new methods for vector control. Studying neuropeptide receptors in particular helps to unravel the neurocrine control of these uncharacterized regulatory mechanisms. The current study set out to examine the potential physiological roles of PK signaling in a vector mosquito by first examining the expression profiles of two PK receptors in different organs of adult *A. aegypti*. We then investigated whether the *A. aegypti* PK1-R and PK2-R identified previously (Choi et al., 2013) are activated by the *AAEL005444* (*capa*) gene-derived PK1 (*Aedae*CAPA-PK1), since this particular pyrokinin was not previously examined. Our current results along with the ability of PKs to stimulate hindgut motility in other insects (Holman et al., 1986) prompted us to further investigate the potential that these neuropeptides may influence myotropic and ionomodulatory activity in the hindgut, as these critical processes contribute toward maintenance of hydromineral balance in nectar- and blood-fed female mosquitoes.

## Materials and Methods

### Animal Rearing

*Aedes aegypti* eggs (Liverpool strain) oviposited onto Whatman filter papers (GE Bioscience) were collected and hatched in plastic containers with distilled water, as previously described (Rocco et al., 2017). Larvae and pupae were reared in a 26°C incubator under a 12:12 h light:dark cycle. Larvae were fed daily with 2% brewers yeast:beef liver (1:1) powder solution (NOW foods, Bloomingdale, Illinois). All adult mosquitoes were fed 10% sucrose (w/v) *ad libitum*, and females in the colony cages were regularly fed with sheep blood in Alsever’s solution (Cedarlane Laboratories, Burlington, ON, Canada) for egg production to maintain the colony. All experiments were performed on 4-day old female and male mosquitoes that were isolated during the pupal stage and transferred into glass mesh-covered jars.

### Receptor Expression Profiles and Pyrokinin Immunolocalization

#### Tissue Dissections, RNA Isolation, cDNA Synthesis and RT-qPCR

Female (*n* = 20) mosquitoes were immobilized with brief exposure to CO_2_, and submerged in nuclease-free Dulbecco’s phosphate-buffered saline (DPBS; Wisent Inc., St. Bruno, QC, Canada). The midgut, Malpighian tubules, pyloric valve (midgut-hindgut junction), ileum, rectum, and reproductive organs (ovaries with accessory reproductive organs, including the common and lateral oviducts, and spermathecae, pooled together) were dissected and transferred into RNA lysis buffer containing 1% 2-mercaptoethanol. Whole-body total RNA samples were obtained from 7-8 females submerged in RNA lysis buffer and homogenized with a plastic microcentrifuge tube pestle and then frozen at −20°C overnight. Total RNA was subsequently extracted using the EZ-10 RNA Miniprep Kit (Bio Basic Inc., Markham, ON, Canada) following the manufacturer’s protocol. The purified RNA was loaded onto a Take3 micro-volume plate and quantified using a Synergy 2 Multi-Mode Microplate Reader (BioTek, Winooski, VT, United States). cDNA was synthesized with 25 ng total RNA as template from each sample using iSCRIPT Reverse Transcription Supermix (Bio-Rad, Mississauga, ON, Canada) following the manufacturer’s instructions and diluted 10-fold for subsequent qPCR analysis.

The synthesized cDNA from mosquito organs was used to assess PK1-R and PK2-R transcript expression by amplifying a 249-bp and 203-bp fragment, respectively, with the forward (5′-TGTACGCTCTGATTGGCCTGAA-3′; PK1-R) or (5′-TATTGTACTTTCTGTCGACGTGC-3′; PK2-R) and reverse (5′- GCACTAATGGATCGTTCGGCTG-3′; PK1-R) or (5′-ATTTGCACCCGTTTTGAAGGAG-3′; PK2-R) primer sets based on a previously identified sequence (Choi et al., 2013; GenBank accession: EAT35008.1, PK1-R; KC155994.1, PK2-R) using PowerUP SYBR Green Master Mix (Applied Biosystems, Carlsbad, CA, United States). A 214-bp fragment of the *rp49* (GenBank accession: AY539746) gene was also amplified as a reference control using primers described previously (Paluzzi et al., 2014) with all cycling conditions as follows: (1) 50°C for 2 min, (2) 95°C for 20 s, and (3) 40 cycles of (i) 95°C for 3 s and (ii) 62°C for 30 s. Relative expression levels were determined using the ΔΔC_*T*_ method and normalized to the *rp49* reference gene. Expression profiles were determined using three biological replicates, each of which included four technical replicates.

#### Enzyme-Linked Immunosorbent Assay (ELISA) and Whole Mount Immunohistochemistry

A custom synthesized rabbit polyclonal antibody (*Rhopr*CAPA-2 antigen sequence: EGGFISFPRV-NH_2_, generously provided by Prof. Ian Orchard, University of Toronto Mississauga, ON, Canada) and recently used to localize CAPA (PRV-NH_2_) immunoreactivity in *A. aegypti* (Sajadi et al., 2020), was used herein to visualize PRXa-like immunolocalization (X = V or L) along mosquito tissues as this antibody was believed to cross-react with structurally related peptides. To confirm that the antibody recognizes PKs, a competitive ELISA was performed. In brief, 96-well plates were coated with anti-*Rhopr*CAPA-2 primary antiserum diluted to 1:1000 in carbonate buffer (15 mM Na2CO3-H2O and 35 mM NaHCO3 in water; pH 9.4) and incubated overnight at 4°C. Plate contents were discarded, blotted, and rinsed three times with wash buffer (350 mM NaCl, 2.7 mM KCl, 1.5 mM KH2PO4, 5.15 mM Na2HPO4-H2O, and 0.05% Tween-20 (v/v) in water). Wells were then incubated for 1.5 h on a rocker with block buffer [0.5% skim milk powder (w/v) and 0.5% BSA (w/v) in PBS]. The block solution was discarded, and standard solutions were added (100 μL/well). Standards consisted of *Aedae*CAPA-1 and *Aedae*CAPA-2 diluted in block buffer to achieve concentrations ranging from 250 pM to 250 nM, or *Aedae*CAPA-PK1 and *Rhopr*PK2b from 4.8 nM to 75 uM. After a 1.5 h incubation on a rocker, 1 nM biotinylated-*Drome*CAPA diluted in block buffer (MacMillan et al., 2018) was added (100 μL/well) to compete with the unlabeled peptide standards for antibody binding. Following overnight incubation at 4°C, the wells were washed four times with wash buffer, and incubated at 4°C for 1.5 h with Avidin-HRP (1:2000; Bio-Rad, Mississauga, ON, Canada). The wells were washed three times, and incubated with 3,3′,5,5′-tetramethylbenzidine (TMB) substrate (100 μL/well; Sigma-Aldrich, Oakville, ON, Canada) for 15 min at RT for color development. Reactions were stopped with 100 μL/well 2N HCl and absorbance at 450 nm was measured using a Synergy 2 Multi-Mode Microplate Reader (BioTek, Winooski, VT, United States).

In light of the PK receptor transcript expression profile and antibody validation confirming pyrokinin cross-reactivity, the pyloric valve region separating the midgut and hindgut organs, as well as the hindgut, including both the ileum and rectum of 4-day old adult female mosquitoes were used to examine PRXa-like immunoreactivity, following a procedure described previously (Rocco et al., 2017). Tissues were incubated in primary antibody solution (diluted 1:1000) made up in 0.4% Triton X-100, 2% normal sheep serum (NSS) (v/v) and 2% BSA (w/v) in PBS. Negative controls involved primary antibody solution pre-incubated with 5 μM *Aedae*CAPA-PK1. Both experimental and control antibody solutions were prepared and left at 4°C overnight prior to incubating with tissues. Following a 48-h primary antibody incubation at 4°C with gentle agitation, tissues underwent three 10-min washes with PBS, and were then incubated with Alexa Fluor 568-conjugated AffiniPure goat anti-rabbit secondary antibody (1:200 dilution; Life Technologies) and 0.165 μM Alexa Fluor 488-conjugated phalloidin (Life Technologies) in 10% NSS (v/v) made up in PBS overnight at 4°C with gentle agitation. Tissues were then rinsed three times with PBS and mounted on slides with mounting media containing 4′,6-diamidino-2-phenylindole dihydrochloride (DAPI) to visualize cell nuclei in tissue preparations. Images were analyzed using a Lumen Dynamics X-Cite^TM^ 120Q Nikon fluorescence microscope (Nikon Instruments Inc., Melville, NY, United States) and a Zeiss Cell Observer Spinning Disk Confocal Microscope (Carl Zeiss Microscopy GmbH, Jena, Germany).

### Heterologous Functional Receptor Assay

#### Preparation of Mammalian Expression Constructs With *A. aegypti* PK1-R and PK2-R

The complete open reading frame was amplified based on the partial (Nene et al., 2007) and complete sequences (Choi et al., 2013) reported earlier for the *A. aegypti* PK1-R (Genbank accession: EAT35008.1) and PK2-R (Genbank accession: KC155994.1). Forward 5′-ATGTTCAGTACAAACCTAAC-3′ (PK1-R) or 5′-ATGATGGAGCTGCAGCAGGTGTCA-3′ (PK2-R) and reverse 5′-TTAATGACGTACCTTGAAAGCTTG-3′ (PK1-R) or 5′-TCAGCGAATCTCATTGTTGATTTCGGCC-3′ (PK2-R) primers were designed over the start and stop codons (underlined), respectively, and used to amplify the complete coding sequence using Q5 High-Fidelity DNA polymerase following manufacturer recommendations (New England Biolabs, Whitby, ON). The 1125 bp (PK1-R) and 1917 bp (PK2-R) PCR products were purified using a Monarch PCR purification kit (New England Biolabs, Whitby, ON, Canada) and reamplified using the identical reverse primers but forward primers possessing the consensus Kozak translation initiation sequence (Kozak, 1984; Kozak, 1986), 5′-GCCACCATGTTCAGTACAAACCTAAC-3′ (PK1-R) or 5′-GCCACCATGATGGAGCTGCAGCAGGTGTCA -3′ (PK2-R). The resulting products were cloned into pGEM-T Easy sequencing vector, and miniprep samples were sequenced to verify base accuracy. The receptor constructs were excised using standard restriction enzyme digestion and subcloned into the mammalian expression vector, pcDNA 3.1^+^ (Life Technologies, Burlington, ON, Canada). Transfection quality plasmid DNA was purified from an overnight bacterial culture using the PureLink MidiPrep Kit (Invitrogen, Burlington, ON, Canada) following manufacturer guidelines.

#### Cell Culture, Transfections, and Bioluminescence Assay

Chinese hamster ovary cells (CHO-K1) described previously (Paluzzi et al., 2012; Gondalia et al., 2016; Wahedi and Paluzzi, 2018) were grown in Dulbecco’s modified eagles medium: nutrient F12 (DMEM:F12) media containing 10% heat-inactivated fetal bovine serum (FBS; Wisent, St. Bruno, QC, Canada), 200 μg/mL geneticin, and antimycotic-antibiotic mixture as described previously (Wahedi and Paluzzi, 2018). Cells were grown to approximately 80% confluency and were co-transfected with mammalian codon-optimized aequorin using Lipofectamine 3000 transfection reagent following recommended guidelines (Invitrogen, Burlington, ON, Canada) to transiently express either the *A. aegypti* PK1-R or PK2-R. Cells were then prepared for the functional assay that was performed 48 h post-transfection following a protocol described previously (Wahedi and Paluzzi, 2018), at which point mCherry-expressing cells showed a transfection efficiency of about 90%. Various concentrations of synthesized PK peptides and other peptides (purity > 90%; Genscript, Piscataway, NJ, United States) were prepared in BSA media and loaded in quadruplicate into white 96-well luminescence plates (Greiner Bio-One, Germany).

Luminescence responses to *A. aegypti* CAPA-1 (GPTVGLFAFPRV-NH_2_), CAPA-2 (pQGLVPFPRV-NH_2_) and CAPA-PK1 (AGNSGANSGMWFGPRL-NH_2_) peptides, along with the *R. prolixus* PK2 orthologs, PK2a (NTVNFSPRL-NH_2_), and PK2b (SPPFAPRL-NH_2_) were examined. A list of peptides used in this study and sequence comparison to native *A. aegypti* pyrokinin peptides is found in Supplementary Table S1. Cells prepared for the functional assay were loaded into each well of the plate using an automated injector unit and luminescent response was measured with a Synergy 2 Multi-Mode Microplate Reader (BioTek, Winooski, VT, United States). Negative controls were carried out using BSA media alone whereas 50 μM ATP, which activates endogenously expressed purinoceptors (Iredale and Hill, 1993; Michel et al., 1998), was used as a positive control. Luminescence responses were normalized to ATP responses and analyzed in GraphPad Prism 7.02 (GraphPad Software, San Diego, CA, United States). EC_50_ values were determined using dose-response curves from multiple biological replicates.

### Hindgut Contraction Assays

#### Preparation of Hindgut Tissues

Hindgut contraction assays were conducted on isolated ilea and recta of female *A. aegypti*. Mosquitoes were anesthetized with CO_2_ and dissected under saline, containing 150 mM NaCl, 25 mM N-2-hydroxyethylpiperazine-N′-2-ethanesulfuronic acid (HEPES), 3.4 mM KCl, 7.5 mM NaOH, 1.8 mM NaHCO_3_, 1 mM MgSO_4_, 1.7 mM CaCl_2_-2H_2_O and 5 mM glucose, titrated to pH 7.1. The isolated tissue was secured with minutien pins within a Sylgard-lined petri-dish.

#### Peptide and Neurotransmitter Dosages

Given its high sequence similarity to endogenous *A. aegypti* PK2 peptides (Supplementary Table S1), we used the *R. prolixus* PK2b peptide (Paluzzi and O’Donnell, 2012), henceforth referred to as *Rhopr*PK2b, for motility bioassays. Commercially synthesized peptides (Genscript, Piscataway, NJ, United States), *Aedae*CAPA-PK1 (AGNSGANSGMWFGPRL-NH_2_) and *Rhopr*PK2b (SPPFAPRL-NH_2_), were used to examine myomodulatory activity. Serotonin (5-hydroxytryptamine, 5-HT; Sigma-Aldrich, Oakville, ON, Canada), previously shown to stimulate *A. aegypti* hindgut contractions (Messer and Brown, 1995), was used as a stimulatory control. Given the inhibitory properties of myoinhibitory peptides (having the consensus W(X_6_)W-NH_2_ carboxyl terminus) on insect hindgut motility (Lange et al., 2012), *Rhopr*MIP-7 (AWNSLHGGW-NH_2_; Genscript, Piscataway, NJ, United States; Paluzzi et al., 2015) was used as an inhibitory control. All hormones were diluted in saline to achieve a final concentration of 1 μM.

#### Electrophysiological Measurements of Recta

To assess whether PK1 peptides plays a role in regulating rectal motility, contractions were monitored in saline to obtain baseline contraction rates and subsequently following *Aedae*CAPA-PK1 or 5-HT treatments, using probes connected to an impedance converter (UFI model 2991, Morro Bay, CA, United States) connected to a Powerlab 4/30 and laptop computer running LabChart Pro 6.0 software (AD Instruments, Colorado Springs, CO, United States). The isolated rectum was contained in a small circular ridge of a Sylgard-coated dish which was bathed in saline. Probes were positioned on either side of the rectum by observation with a dissecting microscope (Olympus SZ61), and contractile responses were recorded on LabChart Pro 6.0 software (AD Instruments, Colorado Springs, CO, United States). Contractions were monitored for two minutes prior to, and after the addition of either *Aedae*CAPA-PK1 or 5-HT.

The number of contractions were recorded over a 2-min interval to obtain the contraction frequency (number of contractions min^–1^). To account for variability in myoactivity between individual preparations, rectal myoactivity is expressed as a ratio in contraction frequency upon *Aedae*CAPA-PK1 or 5-HT application relative to the baseline contractile activity of the same tissue preparation in saline alone.

#### Video Measurements of Ilea

Unlike the rectum, the mosquito ileum produces weaker contractions that we were unable to measure using an impedance converter as described above. As a result, to examine the effects of a PK2 peptide on anterior hindgut motility, video recordings of dissected ilea were obtained using an Olympus SZ microscope connected to Luminera’s INFINITY1-2CB video camera. The dissected gut was pinned in the midgut and rectum to allow the ileum to freely contract in saline and following treatments. Contractions were recorded for 2 min in saline that was used for baseline activity measurements, followed by three subsequent 2-min recordings, including: (i) additional saline, (ii) 1 μM *Rhopr*PK2b, and (iii) either 1 μM 5-HT with *Rhopr*PK2b or 1 μM *Rhopr*MIP-7 with *Rhopr*PK2b. The *Rhopr*PK2b was added along with the stimulatory or inhibitory compound (5-HT or *Rhopr*MIP-7, respectively), to maintain its 1 μM concentration in the bath.

Using the video recordings, the contraction rate (number of contractions min^–1^), average duration of each contraction (sec), and average length of time between each contraction (sec) over the 2-min interval were recorded individually. To account for any potential changes in tissue contractile activity upon adding solutions to the bath, each of these variables were measured relative to baseline saline recordings of the same preparation as a ratio of change, where a value above or below one indicates an increase or a decrease, (respectively), in the rate, duration or length between each contraction in response to the treatment.

### Ion Transport Along the Rectal Pad Epithelia

#### Preparation of Hindgut Tissues

Female mosquitoes were anesthetized on ice for 3 min and dissected in Ca^2+^-free *A. aegypti* saline, as described previously (Paluzzi et al., 2014), to limit spontaneous hindgut contractions during ion flux measurements. The isolated rectum was then transferred to a Petri dish with saline, pre-coated with poly-L-lysine (Paluzzi et al., 2014) to allow the tissue to adhere to the bottom of the dish.

#### Peptide Dosages and Saline Application

*Aedae*CAPA-PK1 or *D. melanogaster Droso*-leucokinin (NSVVLGKKQRFHSWG-NH_2_, Genscript, Piscataway, NJ, United States; provided by Prof. Dick Nässel, Stockholm University, Sweden) were solubilized in double distilled water as a 1 mM stock and then diluted in the above-mentioned Ca^2+^-free *A. aegypti* saline to achieve a 1 μM final concentration. Control measurements were obtained by applying an equal volume of saline only, referred to as saline control treatments.

#### Scanning Ion-Selective Electrode Technique (SIET)

To measure Na^+^ flux across rectal pad epithelia, ion-selective microelectrodes and reference electrodes were used, as described previously (Paluzzi et al., 2014), with the following changes: the microelectrode was backfilled with 100 mM NaCl, front loaded with Na^+^-selective ionophore (sodium ionophore II cocktail A; Fluka, Buchs, Switzerland), and calibrated before every preparation with 200 mM NaCl, and 20 mM NaCl containing 180 mM LiCl to equalize osmolarity of the standard solutions.

SIET measurements were obtained through the Automated Scanning Electrode Technique (ASET) software (Science Wares, East Falmouth, MA, United States). To obtain background recordings for every preparation, the microelectrode tip was positioned at a site located 3 mm away from the tissue. Voltage gradients were measured as the microelectrode moved perpendicularly to the tissue surface between two points separated by a distance of 100 μm. The sampling protocol used a wait time of 4 s after microelectrode movement and a recording time of 1 s after the wait period. Following background voltage readings, the ion-selective microelectrode tip was positioned at a distance of 2 μm from the rectal pad epithelia. A similar sampling protocol was used at the tissue surface. For each sample, several initial measurements were obtained at various sites across the length of one rectal pad and the site demonstrating maximal ion flow was used for all subsequent measurements. Specifically, the sampling protocol was repeated six times at this target site in saline solution, and the voltage difference between the two sites was used to calculate a voltage gradient by the ASET software. A treatment (either saline or peptide) was then directly applied to the dish to determine if this induced a change in ion flux by the rectal pad epithelia. The sampling protocol was then repeated 12 times. Following measurements at the rectal pad sites, background voltage readings were again recorded at a distance of 3 mm away from the tissue.

Calculation of ion flux used in this study has been described previously (Paluzzi et al., 2014). Approximately 70% of tissues initially exhibited Na^+^ absorption in saline, whereas the remaining preparations (approximately 30%) were initially secreting Na^+^ into the rectal lumen. In light of this potential source of variation, further experimental treatment was only carried out on preparations exhibiting hemolymph-directed ion transport (i.e. reabsorbing Na^+^). The change in flux upon treatment application was calculated by subtracting flux values obtained during saline measurements, in which a positive change indicates increased ion absorption, and a negative change represents a decrease in absorption.

### Graphical Representation

Data were transferred into GraphPad Prism 7.0 to create all figures and conduct statistical analyses, which are described as appropriate in the figure captions.

## Results

### Receptor Expression Profile and Localization of PRXa-like Immunoreactivity

**FIGURE 1:**
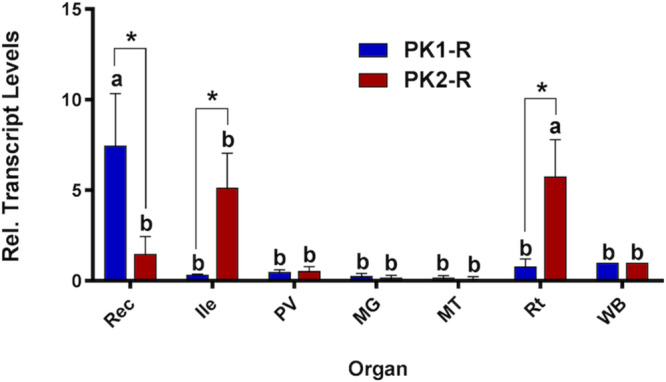
Spatial expression patterns of PK1-R and PK2-R transcript in female organs relative to the whole body (WB), normalized to the reference gene *rp49*. Expression was analyzed in the rectum (Rec), ileum (Ile), pyloric valve (PV) region, midgut (MG), Malpighian tubules (MT) and reproductive organs (Rt). Levels significantly different from the WB are denoted with different letters, and significant differences in transcript abundance between the two receptors in an individual organ are denoted by an asterisk, as determined by a two-way ANOVA and Sidak post test (*p* < 0.05). Data represent the mean ± SEM (*n* = 3).

As a first step toward discovering physiological roles for pyrokinins in *A. aegypti*, prospective targets were examined. RT-qPCR was used to measure PK1 and PK2 receptor transcript levels in adult organs. Expression of PK1-R was only significantly enriched in the rectum compared to expression in the whole body (Figure 1). Comparatively, PK2-R was abundant in the anterior ileum and significantly enriched in reproductive organs relative to the whole body (Figure 1), and demonstrated significantly higher expression compared to PK1-R levels in these organs, which was consistent across all biological replicates.

**FIGURE 2:**
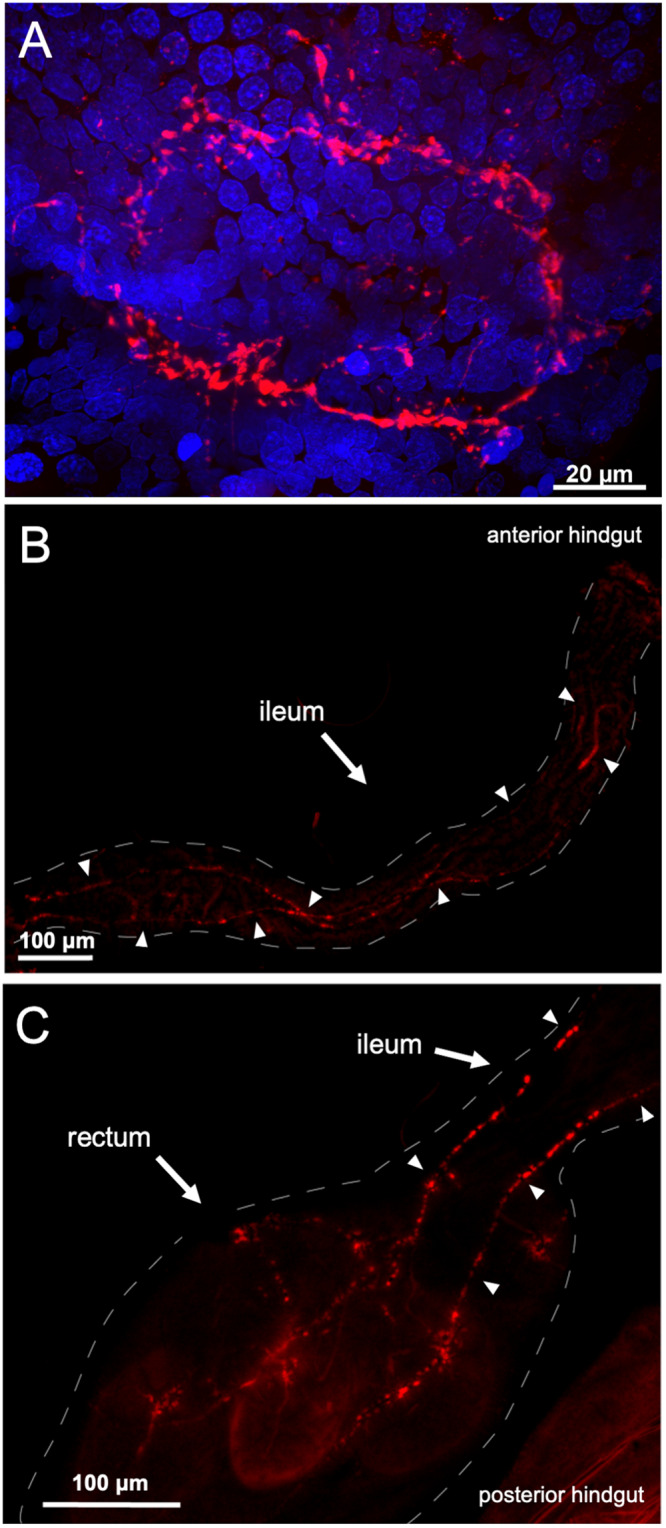
PRXa-like immunolocalization associated with the female gut. Immunoreactive staining (red) was observed within an axon net encircling the pyloric valve at the junction between the midgut and hindgut with nuclei (blue) stained with DAPI **(A)**. PRXa-like immunoreactive axonal projections continue over the anterior hindgut, denoted by arrowheads **(B)** and terminate within the lumen of the six rectal pads **(C)**.

Given this highly defined expression within the two segments of the hindgut, we next used immunohistochemistry to visualize peptide distribution along this region. Using a custom-synthesized antibody against a CAPA neuropeptide, which was shown to cross-react with and bind to PKs (Supplementary Figure S1), PRXa-like immunostaining was observed in an axon net encircling the pyloric valve (Figure 2A), which separates the midgut and hindgut. Immunolocalization was detected in axonal projections over the ileum (Figure 2B), and innervating the rectal pads with immunoreactive projections terminating in close association and encircling 4-5 cells within the lumen of all six rectal pads (Figure 2C; Supplementary Figure S2). Staining was abolished in control preparations treated with antibody pre-incubated with *Aedae*CAPA-PK1 (Supplementary Figure S3).

### PK1-R and PK2-R Functional Activation Assay

**FIGURE 3:**
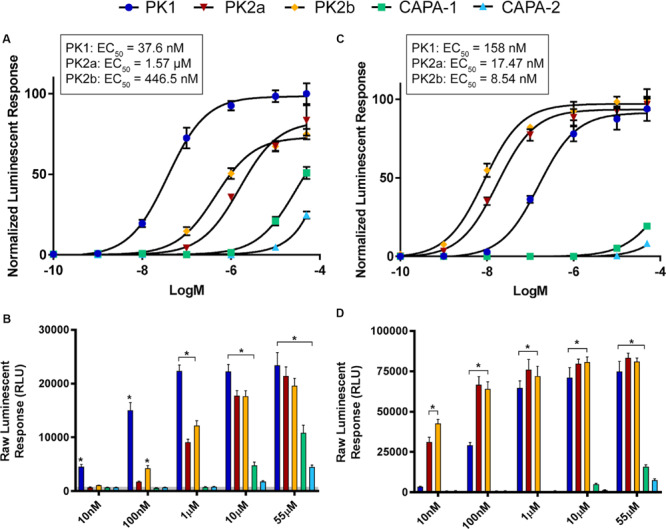
Luminescent response of CHO-K1 cells expressing the *A. aegypti* PK1 **(A,B)** and PK2 **(C,D)** receptors. Transient expression of *A. aegypti* PK1-R in CHO-K1 cells was used to examine receptor functional activation by *Aedae*CAPA-PK1 (denoted as PK1) demonstrating a dose-dependent luminescent response following peptide application **(A,C)**. Structurally related peptides, including two exogenous PK2 analogs (denoted as PK2a and PK2b) along with endogenous *AAEL005444* gene-derived CAPA anti-diuretic peptides (denoted CAPA-1 and CAPA-2) demonstrated significantly lower activity on the heterologously expressed *A. aegypti* PK1 receptor **(B)**. Although *Aedae*CAPA-PK1 was able to activate both receptors, PK2-R displayed more selective activation by the PK2 analogs, PK2a and PK2b, derived from the *R. prolixus ADA83379.1* gene **(C,D)**. Luminescent responses were monitored after peptide treatment, with data representing average luminescence (mean ± standard error) over the first 10 s immediately following peptide application. Raw luminescent responses significantly different from background responses (BSA media alone shown in the gray shaded region) are denoted by an asterisk, as determined by a one-way ANOVA and Dunnett’s multiple comparison *post hoc* test (*p* < 0.05). Data obtained from two individual biological replicates, each consisting of 4-8 technical replicates. Sequences of the tested peptides along with other related endogenous peptides are listed in Supplementary Table S1 highlighting the conserved core bioactive sequence within each peptide family.

Heterologous expression and functional analysis of the *A. aegypti* PK1-R revealed a robust activation by *Aedae*CAPA-PK1 (EC_50_ = 37.6 nM), as demonstrated by the dose-dependent luminescent response (Figure 3A) in CHO-K1 cells transiently expressing *A. aegypti* PK1-R. Additionally, the *A. aegypti* PK1-R was responsive to the *R. prolixus* PK2 orthologs, *Rhopr*PK2a and *Rhopr*PK2b (EC_50_ = 1.57 and 0.4465 μM, respectively), encoded by the *R. prolixus ADA83379.1* gene (Jurenka and Nusawardani, 2011), albeit 12- to 42-fold reduced activity compared to *Aedae*CAPA-PK1. Structurally related peptides (*Aedae*CAPA-1 and -2) encoded by *A. aegypti AAEL005444* gene were also effective in activating PK1-R at high concentrations. The efficacy of these other peptides, however, was orders of magnitude lower than *Aedae*CAPA-PK1, which elicited a luminescent response significantly greater than that achieved with all other tested peptides. Notably, at 10 nM, *Aedae*CAPA-PK1 was the only peptide which elicited a significant luminescent response different from controls treated with BSA assay media alone (Figure 3B).

Cells transiently expressing the *A. aegypti* PK2-R (Figure 3C) were significantly more responsive to the PK2 peptides, *Rhopr*PK2a and *Rhopr*PK2b (EC_50_ = 17.47 and 8.54 nM, respectively), particularly at lower doses of 10 nM (Figure 3D) since no other tested peptide at this concentration had a significant effect on *A. aegypti* PK2-R response. Although *Aedae*CAPA-PK1 was still able to activate PK2-R at high concentrations (EC_50_ = 158 nM), it elicited an over four-fold greater potency on PK1-R expressing cells (Figures 3A,C). Similar to results observed in PK1-R functional expression, the structurally related CAPA peptides (*Aedae*CAPA-1 and -2) were only active on PK2-R at very high concentrations and did not achieve over 25% activation relative to the highly potent pyrokinins (Figures 3C,D).

### Pyrokinins on Mosquito Hindgut Motility

**FIGURE 4:**
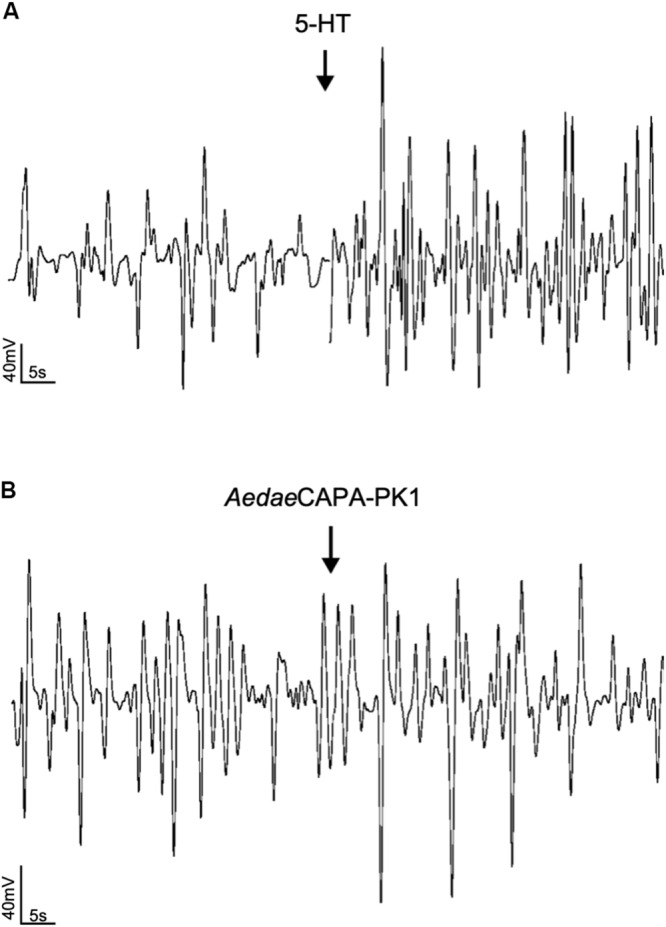
Sample traces of 5-HT **(A)** and *Aedae*CAPA-PK1 **(B)** on spontaneous rectal contractions. Arrows indicate time of hormone application following baseline saline measurement. Contraction rate increased in response to 5-HT **(A)**, whereas no change in activity was observed following *Aedae*CAPA-PK1 application **(B)**.

**FIGURE 5:**
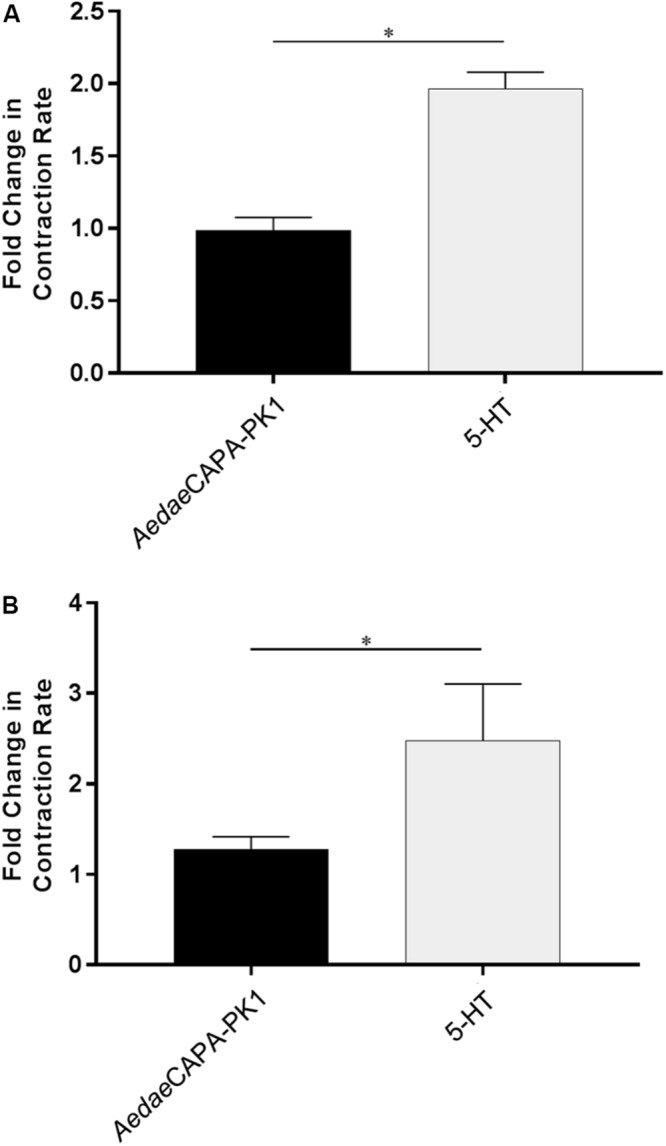
Fold change in contraction frequency of recta isolated from female **(A)** and male **(B)** mosquitoes in response to 5-HT and *Aedae*CAPA-PK1 relative to baseline activity. Mean ± SEM are obtained from 6–18 preparations, with asterisks representing significant differences, as determined using an unpaired *t*-test (*p* < 0.001).

Having verified specific sites of *A. aegypti* PK1-R and PK2-R transcript enrichment and functional activation of these receptors with greatest sensitivity to PK1 and PK2 peptides, we sought to determine the potential myotropic activity of these neuropeptides on the rectum and ileum, respectively. Serotonin (5-HT), a known myostimulator of *A. aegypti* hindgut (Messer and Brown, 1995), was used as a positive control to validate functionality of the bioassay setup. Application of 5-HT typically stimulated greater contractile activity compared to unstimulated baseline activity (Figure 4A) reflecting a 1.97-fold increase in contraction rate for female recta (Figure 5A) and a 2.48-fold increase for male recta (Figure 5B). *Aedae*CAPA-PK1 had no apparent effect on contractile activity (Figure 4B) with no change in the contraction rate (0.99-fold) for female recta (Figure 5A) and an increase (1.28-fold) for male recta (Figure 5B).

**FIGURE 6:**
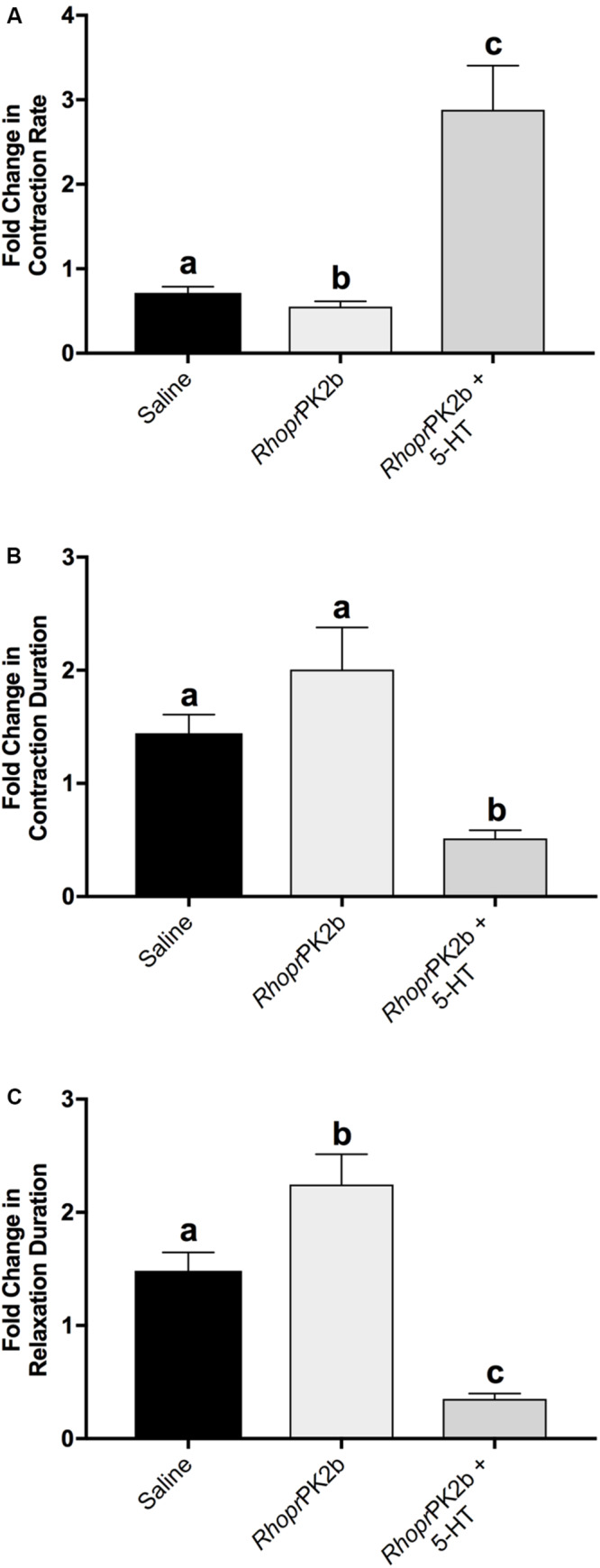
Motility of ilea isolated from female mosquitoes in response to added saline (vehicle control), *Rhopr*PK2b and 5-HT (stimulatory control). The change in contraction frequency **(A)**, duration **(B)** and length of time between contractions **(C)** was measured relative to baseline recordings. Values are presented as mean ± SEM from 15 preparations. Significant differences between the treatments are denoted by different letters, as determined by a one-way repeated measures ANOVA followed by Tukey’s multiple comparison test (*p* < 0.05).

**FIGURE 7:**
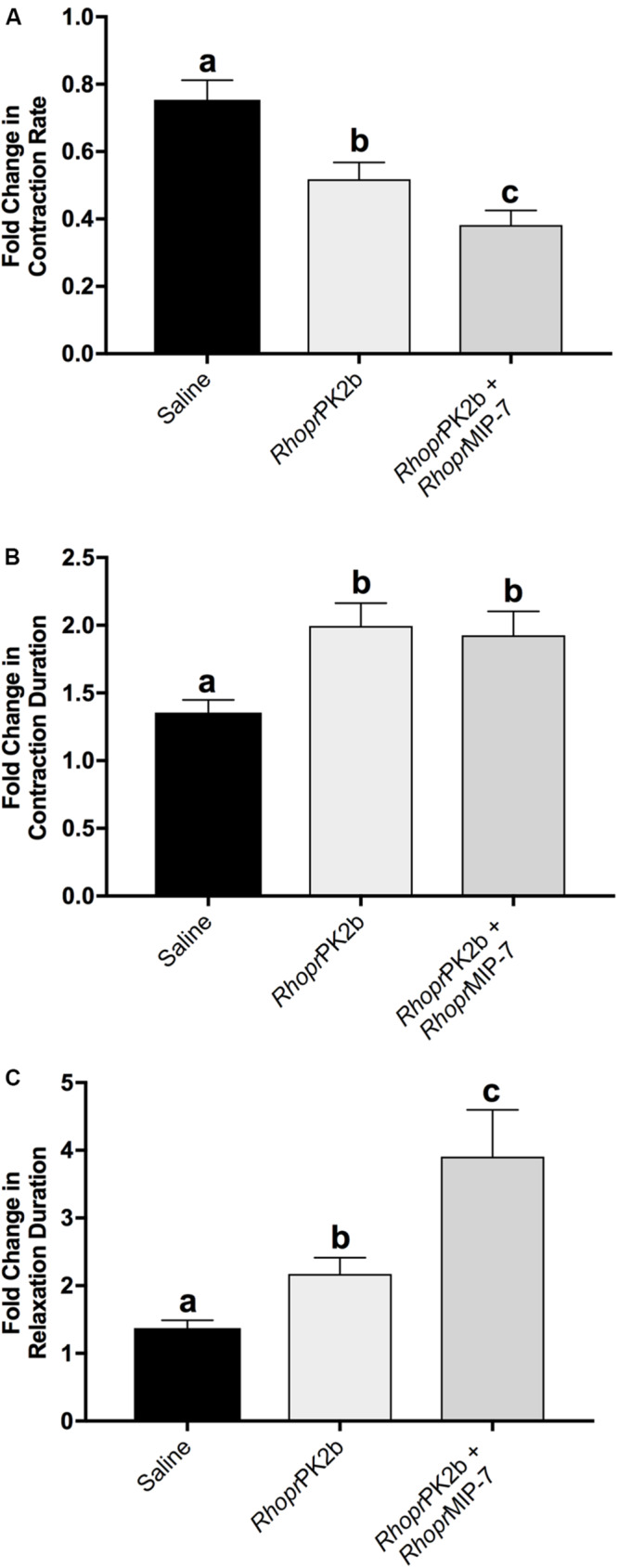
Motility of ilea isolated from female mosquitoes in response to added saline (vehicle control), *Rhopr*PK2b and *Rhopr*MIP-7 (inhibitory control). The change in contraction frequency **(A)**, duration **(B)** and length of time between contractions **(C)** was measured relative to baseline recordings. Values are presented as mean ± SEM from 17 preparations. Significant differences between the treatments are denoted by different letters, as determined by a one-way repeated measures ANOVA followed by Tukey’s multiple comparison test (*p* < 0.05).

To assess the role of PK2 along the anterior hindgut, we used both a stimulatory as well as an inhibitory control following *Rhopr*PK2b treatment. Given the potent bioactivity of *Rhopr*PK2b on the mosquito PK2-R (Figures 3C,D), this naturally occurring analog can be used as a proxy to determine the action of endogenous PK2 peptides in the mosquito. Relative to baseline levels, contraction frequency significantly decreased from 0.72-fold to 0.55-fold in response to *Rhopr*PK2b (Figure 6A; Supplementary Video S1). This inhibitory effect was reversed upon 5-HT treatment. Although the duration of each contraction did not significantly differ upon *Rhopr*PK2b application, the length of time between each contraction event increased. 5-HT effectively reduced both of these metrics, resulting in a 2.88-fold increase in ileal contraction rate (Figures 6B,C). Due to its inhibitory nature, we further assessed the effects of *Rhopr*PK2b with the addition of a known insect myoinhibitor, *Rhopr*MIP-7 (Lange et al., 2012). Although *Rhopr*PK2b significantly reduced ileal contraction frequency, *Rhopr*MIP-7 resulted in further inhibition by 0.38-fold (Figure 7A; Supplementary Video S2). The duration of each contraction increased in response to both peptides by about 1.9-fold (Figure 7B), and *Rhopr*MIP-7 further enhanced relaxation duration by 3.9-fold (Figure 7C).

### *Aedae*CAPA-PK1 on Hindgut Ion Transport

**FIGURE 8:**
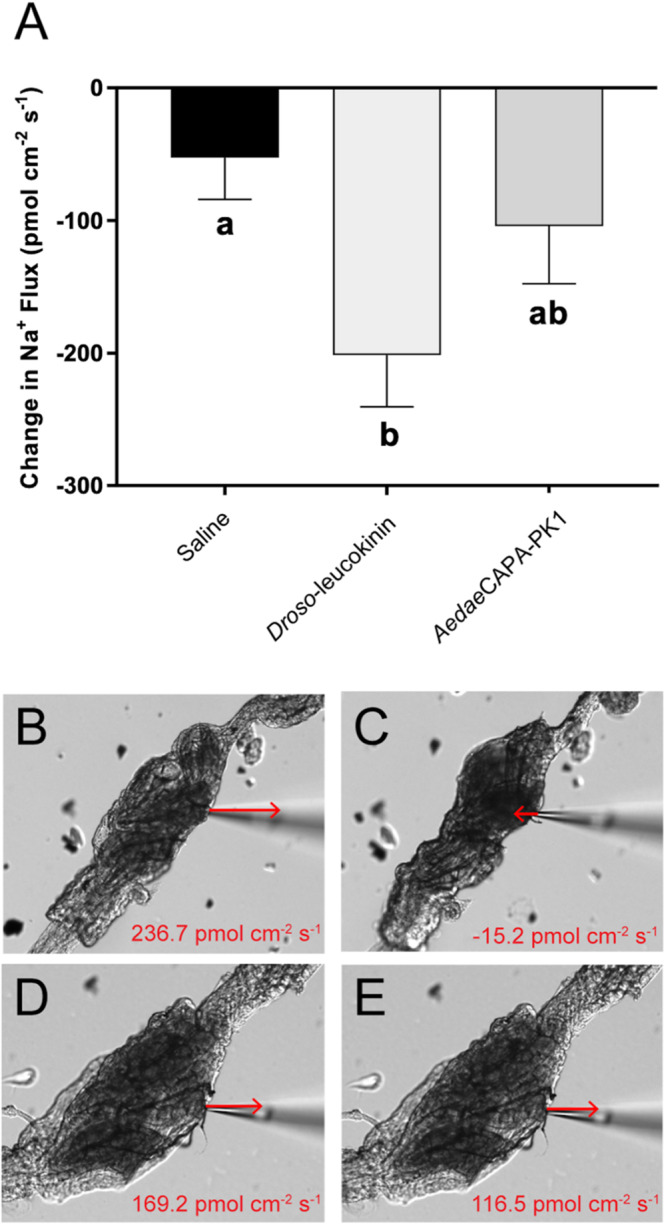
Changes in Na^+^ transport across female rectal pad epithelia in response to saline (vehicle control), *Droso*-leucokinin and *Aedae*CAPA-PK1. Experiments were run only using hindgut preparations that were initially exhibiting hemolymph-directed ion flux (absorption) when unstimulated. Mean ± SEM obtained from 10 to 14 tissue preparations. There was a significant reduction in Na^+^ absorption in response to *Droso*-leucokinin (*p* < 0.05); however, ion transport did not significantly change following *Aedae*CAPA-PK1 treatment in comparison to basal transport activity **(A)**, determined by a one-way ANOVA and Tukey’s *post hoc* test. Still images of the dissected rectum are demonstrated with ion flux recordings obtained from a sample measurement during baseline activity in saline **(B,D)** followed by either *Droso*-leucokinin **(C)** or *Aedae*CAPA-PK1 **(E)** application. Arrows indicate the direction and approximate the magnitude of Na^+^ transport, but are not drawn to scale. Scale bars, 100 μm.

As determined by the SIET, most recta (∼70%) exhibited hemolymph-directed Na^+^ transport (i.e. absorption) in saline prior to treatment, while the remaining recta exhibited lumen-directed transport (i.e. secretion); however, only absorptive rectal preparations were used to examine the potential for *Aedae*CAPA-PK1 in eliciting an ionoregulatory role. Furthermore, measurements of the preparations displayed variability in baseline transport activity in saline, therefore the difference in ion flux, following application of saline alone or containing peptide, relative to initial transport activity in saline was calculated. The resultant data showed that Na^+^ absorption decreased by 52.8 ± 31.4 pmol cm^–2^ s^–1^ after saline application (Figure 8A); however, the net Na^+^ transport remained absorptive. An earlier study showed that the receptor for *A. aegypti* kinins (*Ae*KR) was localized to the hemolymph-facing outer rectal pad membrane and *Ae*KR knockdown by RNA interference decreased excretion (Kersch and Pietrantonio, 2011). This result suggested that an available kinin analog (i.e. *Droso*-leucokinin) may decrease reabsorption over the rectal pads, given that mosquito kinins stimulate diuresis by the Malpighian tubules (Veenstra et al., 1997). Indeed, we found that the ionomodulatory effect of *Droso*-leucokinin along the rectum was significant leading to a four-fold decrease in Na^+^ absorption (201.6 ± 39.0 pmol cm^–2^ s^–1^) compared to saline control (Figures 8A–C). In response to *Aedae*CAPA-PK1, Na^+^ transport decreased two-fold (104.4 ± 43.2 pmol cm^–2^ s^–1^), although this was not significantly different from changes in ion transport following treatment with saline alone (Figures 8A,D,E).

## Discussion

In this study, we have characterized the functional activation of *A. aegypti* PK1 and PK2 receptors in response to various PKs and related neuropeptides. Our findings indicate that PK1-R is most sensitive to PK1 peptides possessing a WFGPRL-NH_2_ carboxyl terminus, also referred to as tryptopyrokinins (Veenstra, 2014), whereas PK2-R is most sensitive to PK2 peptides characterized by their FXPRL-NH_2_ motif. This selective activation was similarly observed in *A. aegypti*, *A. gambiae*, *R. prolixus* and the European corn borer, *Ostrinia nubilalis* (Olsen et al., 2007; Paluzzi and O’Donnell, 2012; Nusawardani et al., 2013; Choi et al., 2013), further confirming the binding specificity and selectivity of PK receptors to their subfamily specific ligands.

Pyrokinins were first discovered in insects based on their effects on hindgut physiology (Holman et al., 1986). The hindgut collects undigested foodstuff passed from the midgut through the pyloric valve, as well as fluid secreted from the Malpighian tubules, and guides these contents along the alimentary canal for waste excretion (Hine et al., 2014). Here we found that relative to other regions examined within the adult mosquito alimentary canal, PK2-R transcript was strongly enriched in the ileum, and PK1-R in the rectum. Our results partially agree with a previous RT-PCR analysis in *A. aegypti* that examined a subset of the tissues/organs we studied herein. Specifically, while enrichment of PK1-R in the midgut and ovaries was not evident as reported earlier (Hellmich et al., 2014), we found this receptor transcript to be significantly enriched in the rectum, similar to findings in other hematophagous arthropods, whereby the tick rectal sac, a structure analogous to the rectum, as well as the ovaries and nervous system were found to express the PK receptor (Yang et al., 2015). For PK2-R, this receptor was previously reported to be expressed within the ovaries in adult female mosquitoes (Hellmich et al., 2014), which is in agreement with our observations showing significant enrichment in female reproductive organs along with the enrichment we observed in the ileum. In contrast, mosquito CAPA receptor expression is highly enriched in the Malpighian tubules, but has no detectable expression in the hindgut (Sajadi et al., 2020). Further, this differential receptor expression supports that PRXa-immunolocalization in this study represents PK peptides, which was much weaker than CAPA-like immunoreactivity localized to neurosecretory cells in abdominal ganglia and associated neurohemal organs (Sajadi et al., 2020), yet the cross-reactivity may also reveal other structurally related peptides.

Due to the extensive network of musculature in the mosquito hindgut (Rocco et al., 2017), as well as the ion transporters dispersed along the epithelia (Patrick et al., 2006), this structure requires the coordination of ionomodulatory and myotropic activity for controlled waste elimination (Kwon and Pietrantonio, 2013). These processes are regulated by neuropeptides along with other neurochemicals and, considering the distinct expression profile of PK receptors in the hindgut that was herein identified along with PRXa-like immunohistochemical staining in association with the hindgut, we examined the potential involvement of pyrokinins in these processes. Specifically, immunostaining was observed in the axon net encircling the pyloric valve that was contiguous with axonal projections over the ileum and extending toward the rectum, revealing these sites as prospective targets for PKs. Although hindgut muscle contractions are myogenic, they can also be modified by neurochemical input from innervation extending from the ventral nerve cord (Audsley and Weaver, 2009). Previously, it was observed that immunostaining for the ovary ecdysteroidogenic hormone (OEH) isolated from *A. aegypti* was associated with the pyloric valve nerve net as well as axonal projections that continued toward the rectum; however, a direct source of this immunoreactivity was not observed although assumed to originate in the ventral nerve cord (Brown and Cao, 2001). Comparatively, cells of the terminal abdominal ganglion innervate the locust hindgut to influence its motility (Donini et al., 2002). Some neurochemical factors have been identified as myotropins acting on *A. aegypti* hindgut, including serotonin and diuretic hormone 31 (Messer and Brown, 1995; Kwon and Pietrantonio, 2013), which also promote fluid secretion across the Malpighian tubules (Sajadi et al., 2018). The concerted action of these and other factors suggest coordination between the diuretic response and hindgut motility to regulate urine and blood bolus expulsion in female mosquitoes.

CAPA-like immunoreactivity was previously identified in pairs of neurosecretory cells of the abdominal ganglia, including the terminal ganglion from which projections that extend onto the hindgut may originate (Sajadi et al., 2020). Given the absence of CAPA receptor transcript in the hindgut (Sajadi et al., 2020), in this study we examined the role of PK1 and PK2 peptides on hindgut motility. The ability of *Rhopr*PK2b to significantly reduce ileal motility suggests endogenous PK2 peptides may have a role in regulating digestive and excretory processes. Visceral muscle contractions along the hindgut aid in the movement of undigested foodstuff following post-prandial diuresis to eliminate waste (Te Brugge et al., 2008). Inhibition of hindgut motility by a PK2 therefore warrants further study to determine its specific function in these processes and during different feeding states. This is the first study to establish a myoinhibitory role for a PK2 peptide on the insect hindgut, since members of this neuropeptide family sharing the conserved FXPRL-NH_2_ carboxyl terminus have previously been characterized as having myostimulatory actions on the hindgut of *L. maderae*, *Periplaneta americana*, *Zophobas atratus*, and *Tenebrio molitor* (Holman et al., 1986; Predel and Nachman, 2001; Marciniak et al., 2012), demonstrating their diverse effects across various insect species.

*Aedae*CAPA-PK1 did not significantly influence myotropic activity in the mosquito rectum, despite receptor transcript enrichment in this organ, which prompted us to examine other potential functions. The rectum serves as the final site for reabsorbing ions, water and essential metabolites back into the hemolymph, ultimately determining the composition of excreted matter (Coast, 2009; Beyenbach and Piermarini, 2011). PRXa-like immunoreactivity revealed axonal projections terminating in close association with cells located within the lumen of the rectal pads (Supplementary Figure S2), structures that protrude from the rectal epithelium. This close association is indicative of a potential neurotransmitter/neuromodulatory role, in which local release at these synaptic terminals may activate receptors on these uncharacterized cells. To date, morphological studies of the mosquito rectum have been limited. Hopkins (1967) was one of the very few to study the ultrastructure of the mosquito rectal pads, revealing a single layer of epithelia surrounding a central canal. The central canal of the rectal pads carries a tracheal trunk, which extends into several tracheolar branches throughout the epithelia. Hopkins (1967) proposed the presence of tracheal and glial cells associated with this tracheal trunk. In some insects, cells along this region have been termed medullary cells, situated in close proximity to neurosecretory terminals (Gupta and Berridge, 1966). In *Blattella* and *Blaberus* rectal pads, axonal projections terminate at sites adjacent to the basal surface of secondary cells, which may help regulate fluid reabsorption (Wall and Oschman, 1973). By examining structural changes in the rectal pads following a blood meal in mosquitoes, these sites have been proposed to play crucial roles in maintaining iono- and osmoregulation to help restore hemolymph homeostasis (Hopkins, 1967). The presence of ion transporters within these structures was later revealed, whereby basolateral P-type Na^+^/K^+^-ATPase and apical V-type H^+^-ATPase staining along the rectal pad epithelia supported that they serve as sites for ion transport, enhancing the overall absorption of ions and water back into the hemolymph prior to waste excretion (Patrick et al., 2006).

Although the exact mechanisms of ion and water transport within the rectal pads have not yet been characterized, the *Ae*KR was previously localized along the hemolymph-facing membrane surface of these structures (Kersch and Pietrantonio, 2011). Our results confirm that a structurally related kinin from *Drosophila*, *Droso*-leucokinin, inhibits Na^+^ absorption at these sites, showing that ion transport mechanisms along the rectal pads may be regulated by neuropeptides. PK1-R transcript detection along with PRXa-like immunoreactivity within the rectum was initially suggestive of an ionoregulatory role at these sites. However, since *Aedae*CAPA-PK1 did not significantly influence Na^+^ transport along the rectal pad epithelia, the function of this peptide on the rectum remains unclear. To better understand its role in mosquito hindgut physiology, it is critical in future studies to identify and characterize the cells closely associated with immunoreactivity within the rectal pad central canal (Supplementary Figure S2), which are distinct from the epithelial cells where *Ae*KR was immunolocalized over the outer rectal pad membrane (Kersch and Pietrantonio, 2011).

The *AAEL005444* gene (Strand et al., 2016) encodes *Aedae*CAPA-PK1 along with two anti-diuretic hormones (*Aedae*CAPA-1 and -2), with the latter regulating the inhibition of fluid secretion across the Malpighian tubules of larval and adult *A. aegypti* (Ionescu and Donini, 2012; Sajadi et al., 2018). In the adult *A. aegypti*, PK-like immunoreactivity has been previously localized in the central nervous system, including numerous cells in the brain, within three groups of neurons of the subesophageal ganglion, and the abdominal ganglia of the ventral nerve cord (Hellmich et al., 2014). In larval stage *A. aegypti*, genes encoding pyrokinins were molecularly characterized revealing *hugin* gene expression, which encodes both PK1 and PK2 neuropeptides, primarily within the subesophageal ganglion, whereas *capa* gene expression was detected mainly within the abdominal ganglia (Hellmich et al., 2014). In support of this observation, neuropeptidomic analyses have shown the presence of pyrokinins originating from both the *hugin* and *capa* gene in the subesophageal ganglion, whereas only *capa* gene-derived *Aedae*CAPA-PK1 was found within the abdominal ganglia, which supply the neurohemal perivisceral organs via the unpaired median nerve (Predel et al., 2010). Given that PK1 and anti-diuretic hormones are derived from a common precursor peptide, along with the proposed roles that rectal pads may play in osmoregulatory processes, there could be some functional relatedness between these peptides, such as binding to distinct receptors expressed along different target organs of the alimentary canal to exert similar overall actions. Localization of PK-like immunoreactivity in close association with cells within the lumen of the rectal pads could suggest a role for *Aedae*CAPA-PK1 in the regulation of ion and water absorption owing to its specific receptor being enriched within this organ. Following nectar or blood feeding, the excess water and ions taken up from the meal pose a challenge to the hydromineral balance of the organism (Coast, 2009). Although *Aedae*CAPA-PK1 did not elicit changes to Na^+^ transport across the rectal pad epithelia of unfed adults, examining other critical processes at these sites, such as anion or water transport, may uncover the role of this neuropeptide in the mosquito rectum. Since the rectal pads have been suggested to play a role in helping to alleviate this insult to hemolymph homeostasis (Hopkins, 1967), *Aedae*CAPA-PK1 may require the initiation of other signaling pathways involved during postprandial diuresis to exert its physiological actions and aid in the regulation of ion and water balance. However, the control of these processes along the hindgut is not yet well understood and, as a result, updating the current model of the *A. aegypti* rectal pad ultrastructure through modern electron microscopy approaches is necessary. This may in turn help unravel the function of *Aedae*CAPA-PK1 that activates its receptor (*Aedae*PK1-R) expressed in this organ, along with other neuropeptides that target these structures.

The presence of *A. aegypti* PK2-R transcript associated with both the ileum and reproductive organs indicate that its PK2 ligands may exhibit pleiotropic actions. Similar to our findings, it was earlier shown by RT-PCR that PK2-R is present in reproductive tissues in *A. aegypti*, where specifically the ovaries were examined (Hellmich et al., 2014), which is consistent with observations on PK receptors in other blood-feeding arthropods, including *R. prolixus, I. scapularis* and the cattle tick, *Rhipicephalus microplus* (Paluzzi and O’Donnell, 2012; Yang et al., 2015; Gondalia et al., 2016). Although their physiological role has not yet been characterized in these hematophagous arthropods, receptor transcript in reproductive organs indicates that PK2 peptides may target these regions to regulate processes critical to mosquito reproduction or development. In other insects, for instance, related PKs were shown to stimulate *L. migratoria, P. americana*, *Z atratus* and *T. molitor* oviduct contractions (Schoofs et al., 1993; Predel and Nachman, 2001; Marciniak et al., 2012), which promote the passage of eggs toward the common oviduct for fertilization (Wigglesworth, 1942). PKs also trigger embryonic diapause by binding to receptors in developing *B. mori* ovaries, upregulating trehalase expression, which promotes glycogen accumulation in oocytes required for the initiation of diapause (Su et al., 1994; Homma et al., 2006; Kamei et al., 2011). Similar roles have been reported in other lepidopteran species, such as the tussock moth, *Orgyia thyellina*, where a PK1 peptide was found to induce embryonic diapause and also promote ovarian development (Uehara et al., 2011).

In *A. aegypti* mosquitoes, females must feed on blood to initiate egg production within the ovaries (Clements, 2000). Upon feeding, neuropeptides such as insulin-like peptides and OEH are secreted from brain neurosecretory cells to stimulate nutrient uptake into developing oocytes by promoting ecdysone synthesis (Brown et al., 1998, 2008; Helbling and Graf, 1998; Riehle and Brown, 1999). PKs have been previously shown to regulate ecdysteroidogenesis upon receptor activation in *B. mori* prothoracic gland (Watanabe et al., 2007). To assess whether PK2 may be involved in similar processes in mosquitoes, future studies should investigate PK2-R expression upon blood feeding to further delineate the involvement of the PK signaling system in regulating these previtellogenic processes. Although no studies to our knowledge have examined the action of PKs on reproductive success in insects, PK2-R transcript expression associated with this organ warrants further investigation to reveal its putative role in mosquito reproductive biology. Given the importance of these PK receptor-enriched organs in a range of physiological activities, these insights may be useful in developing novel strategies to target processes critical to mosquito survival and reproduction, and could ultimately reduce the burden of these disease vectors.

## Data Availability Statement

All datasets generated for this study are included in the article/Supplementary Material.

## Author Contributions

AL performed all the experiments and wrote the initial manuscript. Both authors analyzed the data and contributed towards editing of the final manuscript submitted for publication.

## Conflict of Interest

The authors declare that the research was conducted in the absence of any commercial or financial relationships that could be construed as a potential conflict of interest.
