# Supplementary material for: Receptor Characterization and Functional Activity of Pyrokinins on the Hindgut in the Adult Mosquito, Aedes aegypti
Source: Front Physiol. 2020 May 21;11:490. doi: 10.3389/fphys.2020.00490 (PMC7255104; doi:10.3389/fphys.2020.00490)
Supplement: Supplementary file 3 [file Data_Sheet_1.pdf]

Supplementary Information for:

Receptor Characterization and Functional Activity of Pyrokinins on the Hindgut in the Adult Mosquito, *Aedes aegypti*.

Aryan Lajevardi and Jean-Paul V. Paluzzi*

Laboratory of Integrative Vector Neuroendocrinology, Department of Biology, York University, Toronto, Ontario, Canada, M3J1P3

*** Corresponding author:**
Jean-Paul V. Paluzzi
paluzzi@yorku.ca

This file includes:

Supplementary Table S1
Supplementary Figure S1
Supplementary Figure S2
Supplementary Figure S3
Supplementary Video S1 (caption only)
Supplementary Video S2 (caption only)

Supplementary Table S1. Sequence characteristics of the various peptides used in the study including endogenous *A. aegypti* neuropeptides and homologous peptides from other insects. Peptides in black font were used in this study whereas peptides in blue font are *A. aegypti* peptides listed for sequence comparison. The family-specific C-terminal motifs and conserved residues required for peptide bioactivity are denoted in red font.

Peptide	Sequence	Reference
*Aedae*CAPA–PK1	AGNSGANSGMWFGPRL–NH_2	Predel et al., 2010
*Aedae*PK1	AAAMWFGPRL–NH_2	Predel et al., 2010
*Aedae*PK2	DASSSNENNSRPPFAPRL–NH_2	Predel et al., 2010
*Aedae*PK3	NLPFSPRL–NH_2	Predel et al., 2010
*Rhopr*PK2a	NTVNFSPRL–NH_2	Paluzzi and O'Donnell, 2012
*Rhopr*PK2b	SPPFAPRL–NH_2	Paluzzi and O'Donnell, 2012
*Aedae*CAPA–1	GPTVGLFAFPRV–NH_2	Predel et al., 2010
*Aedae*CAPA–2	pQGLVPFPRV–NH_2	Predel et al., 2010
Droso–leucokinin	NSVVLGKKQRFHSWG–NH_2	Zandawala et al., 2018
Aedae–leucokinin 1	NSKYVSKQKFYSWG–NH_2	Veenstra, 1994
Aedae–leucokinin 2	NPFHAWG–NH_2	Veenstra, 1994
Aedae–leucokinin 3	NNPNVFYPWG–NH_2	Veenstra, 1994
*Rhopr*MIP–7	AWNSLHGGW–NH_2	Paluzzi et al., 2015
*Aedae*MIP1	TWKNLQGGW–NH_2	Kim et al., 2010
*Aedae*MIP2	AWNKINGGW–NH_2	Kim et al., 2010
*Aedae*MIP3	VNAGPAQWNKFRGSW–NH_2	Kim et al., 2010
*Aedae*MIP4	EPGWNNLKGLW–NH_2	Kim et al., 2010
*Aedae*MIP5	SEKWNKLSSSW–NH_2	Kim et al., 2010

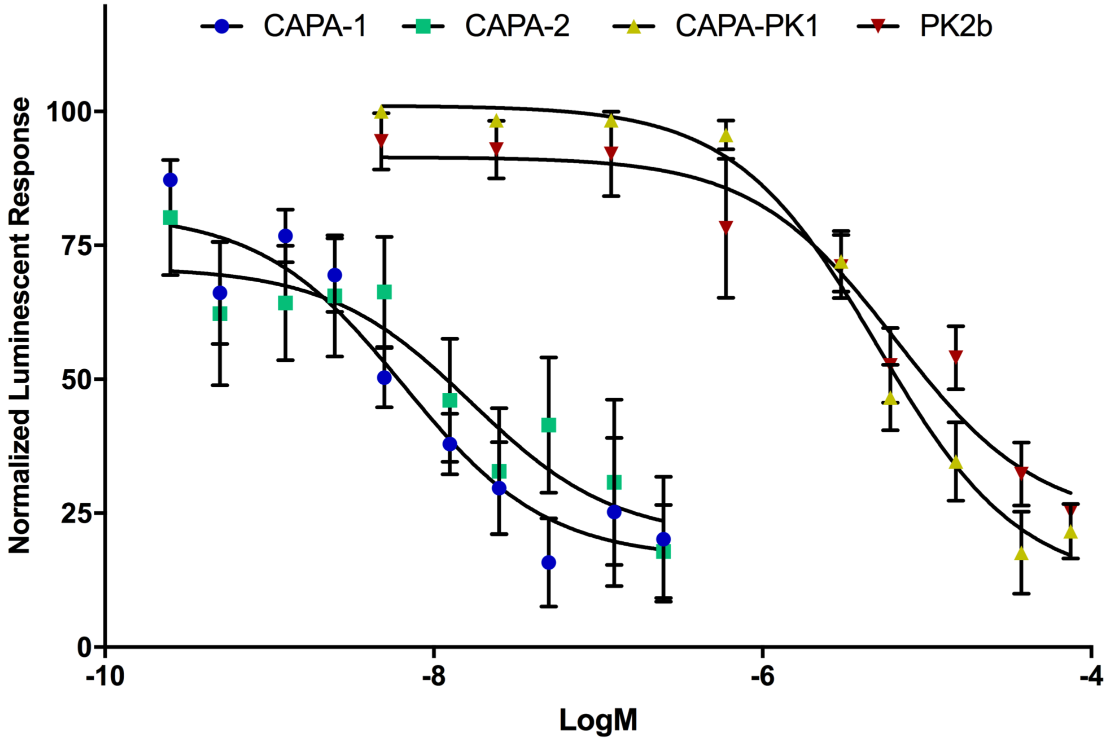

Supplementary Figure S1. Competitive ELISA used to confirm cross-reactivity and binding affinity of CAPA2-targeted antibody to other structurally related peptides. The custom-synthesized antibody designed to target the antigen sequence EGGFISFPRV-NH_2 selectively targets *Aedae*CAPA-1 (IC_{50} = 6.57nM) and *Aedae*CAPA-2 (IC_{50} = 16.93nM), but was also able to recognize and bind pyrokinins, including *Aedae*CAPA-PK1 (IC_{50} = 4.99µM) and *Rhopr*PK2b (IC_{50} = 6.43µM). Normalized responses represent mean ± SEM (n = 4).

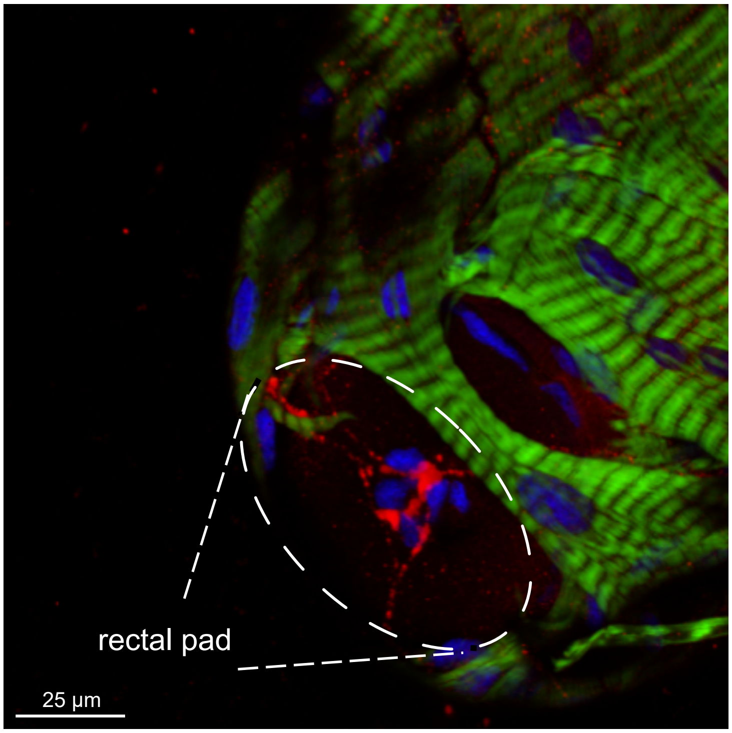

Supplementary Figure S2. PRXa-like immunoreactive processes (red) terminating in close association to cells within the rectal pad (one of six shown) of adult female mosquitoes. No co-localization with phalloidin-stained F-actin (green) was detected.

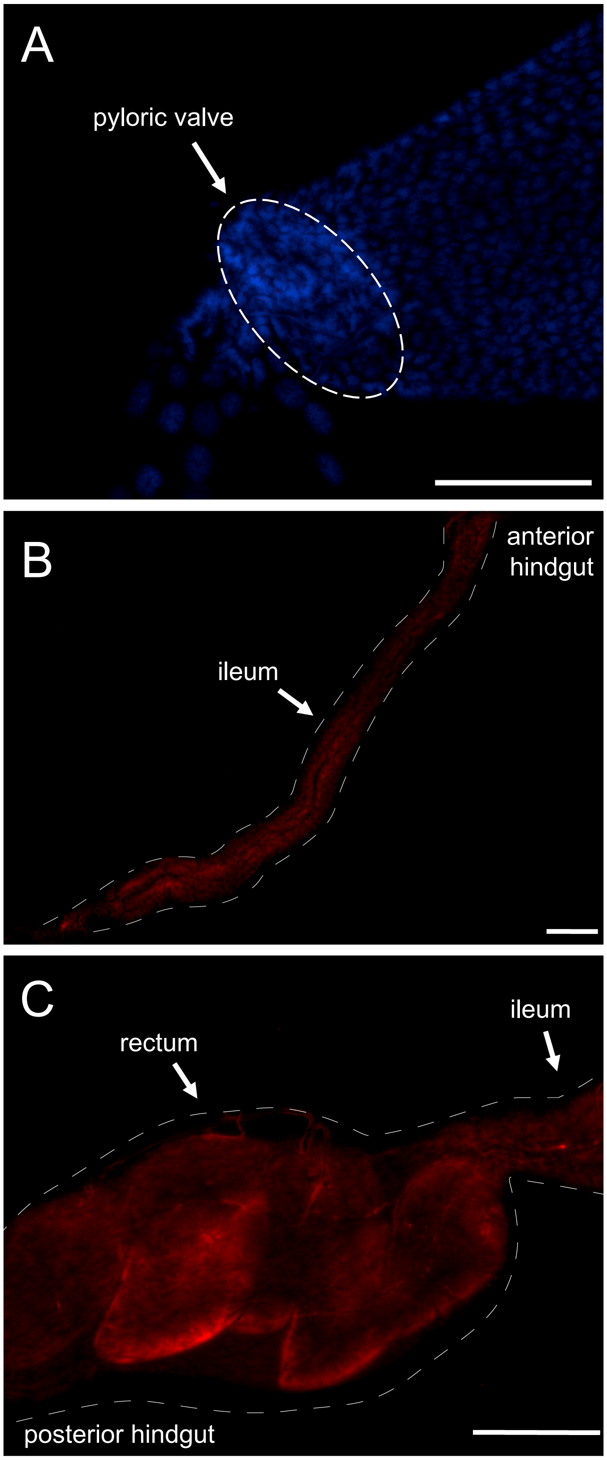

Supplementary Figure S3. Pre-incubation of primary antibody with 5 µM *Aedae*CAPA-PK1 abolishes immunoreactivity along the pyloric valve (**A**), ileum (**B**) and rectum (**C**). All microscope acquisition exposures are equalized to settings used for experimental treatments. Scale bars, 100 µm.

Videos:

Supplementary Video S1. Sample recording of mosquito ileal motility in response to saline (vehicle control), *Rhopr*PK2b and 5-HT (stimulatory control). While *Rhopr*PK2b decreased contraction frequency, 5-HT reversed this effect and notably increased activity above baseline levels. All video speeds are increased to 300%.

Supplementary Video S2. Sample recording of mosquito ileal motility in response to saline (vehicle control), *Rhopr*PK2b and *Rhopr*MIP-7 (inhibitory control). Both *Rhopr*PK2b and *Rhopr*MIP-7 treatments reduced contractile activity of dissected ilea. All video speeds are increased to 300%.

References

Kim, Y.J., Bartalska, K., Audsley, N., Yamanaka, N., Yapici, N., Lee, J.Y., et al. (2010). MIPs are ancestral ligands for the sex peptide receptor. *Proc. Natl. Acad. Sci. U. S. A.* 107, 6520–6525. doi: 10.1073/pnas.0914764107

Paluzzi, J.-P. V., Haddad, A. S., Sedra, L., Orchard, I., and Lange, A.B. (2015). Functional characterization and expression analysis of the myoinhibiting peptide receptor in the Chagas disease vector, *Rhodnius prolixus*. *Mol. Cell. Endocrinol.* 399, 143–153. doi: 10.1016/j.mce.2014.09.004

Paluzzi, J.-P. V., and O'Donnell, M. J. (2012). Identification, spatial expression analysis and functional characterization of a pyrokinin-1 receptor in the Chagas' disease vector, *Rhodnius prolixus*. *Mol. Cell. Endocrinol.* 363, 36–45. doi: 10.1016/j.mce.2012.07.007

Predel., R., Neupert, S., Garczynski, S., Crim, J.W., Brown, M. R., Russell, W.K., et al. (2010). Neuropeptidomics of the mosquito *Aedes aegypti*. J. *Proteome Res.* 9, 2006–2015. doi: 10.1021/pr901187p

Veenstra, J.A. (1994). Isolation and identification of 3 leucokinins from the mosquito *Aedes aegypti*. *Biochem. Biophys. Res. Commun*. 202, 715–719. doi: 10.1006/bbrc.1994.1989

Zandawala, M., Marley, R., Davies, S.A., and Nässel, D.R. (2018). Characterization of a set of abdominal neuroendocrine cells that regulate stress physiology using colocalized diuretic peptides in *Drosophila*. *Cell. Mol. Life Sci*. 75, 1099–1115. doi: 10.1007/s00018-017-2682-y
